# Adolescents and parents’ knowledge of chronic kidney disease: the potential of school-based education

**DOI:** 10.1007/s10157-024-02574-8

**Published:** 2024-10-29

**Authors:** Junko Nakamura, Ryohei Kaseda, Mizuki Takeuchi, Kou Kitabayashi, Ichiei Narita

**Affiliations:** 1 Division of Clinical Nephrology and Rheumatology, Niigata University Graduate School of Medical and Dental Sciences, 1-757 Asahimachi-Dori, Chuo-ku, Niigata, 951-8510 Japan; 2 Department of Health and Nutrition, Faculty of Health Science, Niigata University of Health and Welfare, 1398 Shimami-Cho, Kita-ku, Niigata, 950-3198 Japan; 3 Department of Nutrition, Shinkohkai Murakami Kinen Hospital, 204-1 Matsuyama, Murakami, 958-0034 Japan; 4 Niigata Institute for Health and Sports Medicine, 67-12 Ceigoro Chuo-ku, Niigata, 950-0933 Japan

**Keywords:** Chronic kidney disease, Renal function awareness, Junior high school students, Parental knowledge, Health education

## Abstract

**Background:**

Preventing the progression of chronic kidney disease (CKD), reducing the incidence of new dialysis patients, and increasing public awareness about CKD are pivotal in mitigating renal impairment. This study aimed to assess the relevance of kidney disease and CKD knowledge among junior high school students and their parents.

**Methods:**

A questionnaire survey on kidney function and CKD was conducted among students aged 14–15 years and their parents (851 pairs). Parents were also asked about their age, sex, and participation in health checkups.

**Results:**

The study achieved a collection rate of 49.1%, with a valid response rate of 79.7%. Both junior high school students and their parents exhibited limited knowledge about kidney functions, primarily understanding these functions only in terms of waste product excretion and lacking awareness of other functions. A significant positive correlation was observed in awareness of kidney functions between students and their parents. Regarding CKD awareness, only 2.4% of students and 16.5% of parents were knowledgeable about CKD itself, while 18.9% of students and 45.3% of parents were aware of its name only. Importantly, CKD knowledge among both students and parents was associated, with those aware of CKD also demonstrating better understanding of kidney functions.

**Conclusion:**

This study highlights inadequate knowledge among junior high school students and their parents regarding renal function and CKD. A significant correlation was observed in CKD awareness between students and their parents. These findings underscore the need for targeted strategies to enhance public education and awareness about kidney health.

## Introduction

Chronic kidney disease (CKD) is a major risk factor for cardiovascular events and mortality [1]. CKD affects > 10% of the population worldwide (over 800 million people) and is one of the few non-communicable diseases with an increase in related deaths over the past 20 years [2]. In Japan, the number of patients exceeds 13.3 million [3], and the number of new dialysis patients is estimated to increase by 2030 [4]. Thus, CKD has become a global burden [5].

Internationally, the importance of raising awareness and early detection of CKD has led to the World Kidney Day campaign being held every year since 2006 on the second Saturday of March [6]. This campaign aims to raise awareness of the kidneys and is active in Japan [7]. Activities are also conducted in areas with low health literacy [8]; however, knowledge of CKD remains low worldwide [9–12], hindering prevention and early detection. In Japan, although knowledge of CKD is increasing annually, awareness among the younger generation remains low [13], and effective strategies to promote awareness among this population are needed.

Lifestyle modifications are necessary to treat CKD, diabetes, or hypertension [14]. Lifelong health education through family and school is important for assessing lifestyle-related diseases. The Center for Disease Control and Prevention states that effective strategies for smoking prevention targeting the young generation “include school education [15]”. Education in schools is expected to initiate the transfer of knowledge from students to their families, and the improvement of students’ knowledge can potentially lead to increased awareness of CKD among the younger generations. The transtheoretical model states that knowledge acquisition and understanding are necessary for behavioral changes [16]. The risk of estimated glomerular filtration rate (eGFR) decline is lower after preparation (stage 3) for behavioral changes [17]. Therefore, gaining knowledge about CKD can lead to behavioral changes in the form of lifestyle modifications.

Nevertheless, basic data regarding an increase in knowledge in the younger generation have not been well reported, and few surveys have been conducted on the knowledge of CKD among students who are educated about lifestyle-related diseases. This study aimed to examine the relationship between the knowledge of students and their parents about kidney function and CKD and between parents’ knowledge of CKD and health checkup behavior through a questionnaire survey for junior high school students and their parents.

## Methods

### Study design and participants

This cross-sectional study included 851 junior high school students (aged 14–15 years) from junior high schools in Niigata City and their parents. Eight junior high schools were chosen randomly. Along with the explanation of the survey’s purpose, the document clarified that responses were voluntary, must be submitted anonymously, and were not related to school performance, to ensure that students and parents would not influence each other’s answers. Classroom teachers distributed a single-sided A4-sized questionnaire to the junior high school students. The students and their parents completed the survey at home. Questionnaires were sealed and submitted to a collection box located at each school. This study was conducted from July 7 to 25, 2022.

### Questionnaire related to CKD

**Fig. 1:**
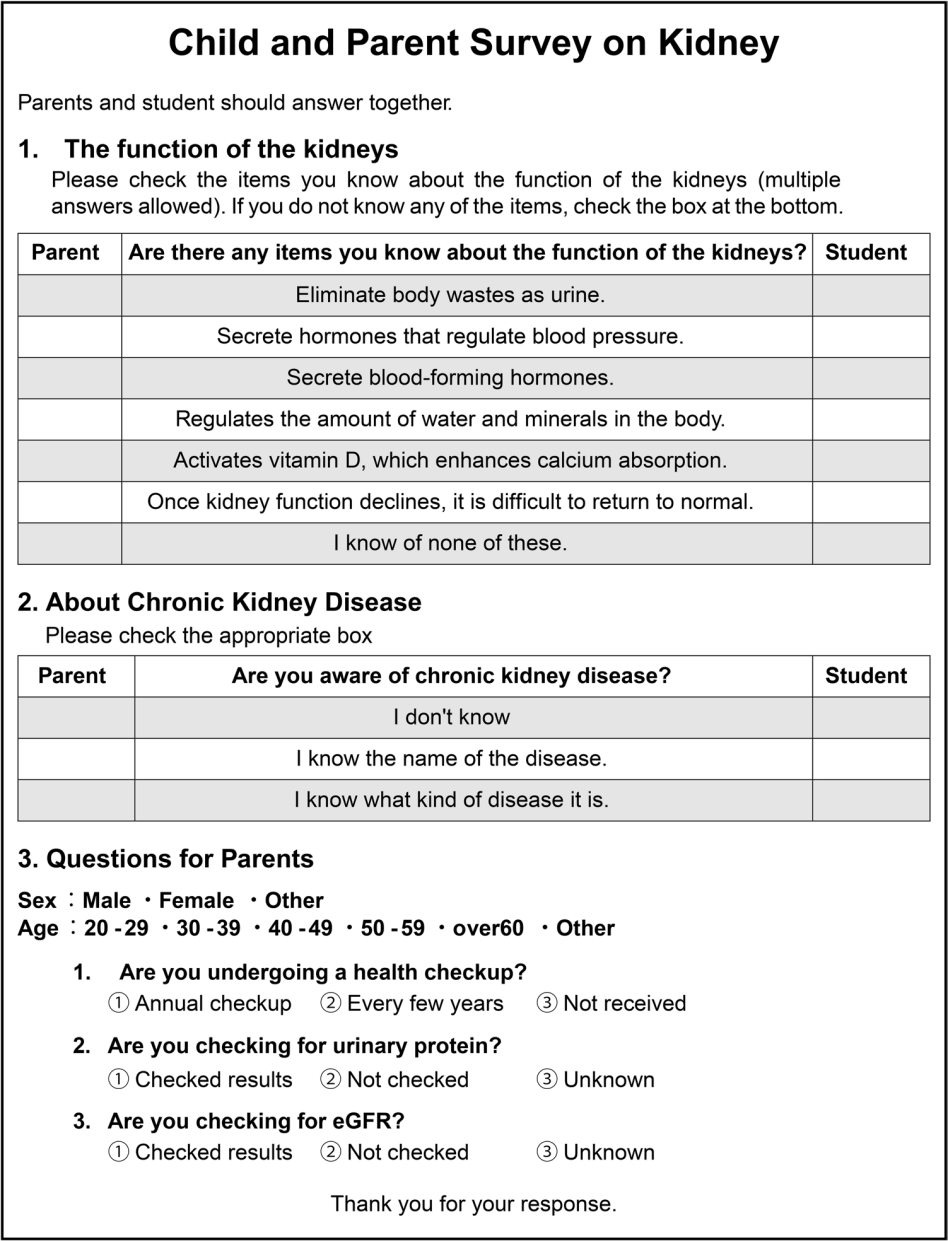
Questionnaire designed for joint completion by students and their parents. Questions 1 and 2 are answered by both the student and parent, whereas Question 3 is answered only by the parent

The questionnaire consisted of three parts (Fig. 1). Part 1 of the questionnaire was related to renal function: Are there any items you know about the function of the kidney? The options were as follows: eliminate body wastes such as urine; secrete hormones that regulate blood pressure; secrete blood-forming hormones; regulate the amount of water and minerals in the body; activates vitamin D, which enhances calcium absorption; once kidney function declines, it is difficult to return to normal; I know none of these. Part 2 was about CKD: are you aware of chronic kidney disease? The options were as follows: I do not know; I know the name of the disease; I know what kind of disease it is. In Part 3, the students’ parents were asked about their sex, age, health checkups received, and urinary protein and eGFR confirmed. Based on the answer in Part 2, the participants were classified into three groups: people who know CKD, people who know only the name, and people who do not know CKD. To analyze the association between students’ and parents’ knowledge of CKD, we considered participants who responded, “I know the name of the disease” or “I know what kind of disease it is” as “people who know CKD.”

### Statistical analysis

The association between the number of cognitive items related to kidney function for students and their parents was analyzed using Spearman’s rank correlation coefficient. Cross-tabulations and Chi-squared tests were conducted to determine the association between students’ and parents’ knowledge levels. Comparisons among the three groups according to CKD knowledge were performed using the Kruskal–Wallis test and the Mann–Whitney *U* test with Bonferroni correction. Cross-tabulation and Chi-squared tests were conducted to determine the association between CKD knowledge and health checkups, confirmed urinary protein levels, and confirmed eGFR. Statistical analyses were performed using SPSS version 26 for Windows (IBM Corp.; Armonk, NY, USA), and the post-hoc power of the effect size was calculated with G*Power 3.1.9.7 software [18] using the significance level (0.05), total sample size (n = 333), and correlation coefficient (ρ) or degrees of freedom (φ, Cramer’s V). Statistical significance was assessed using two-tailed tests with *p* < 0.05.

### Ethical considerations

This survey was conducted in accordance with the Ethical Guidelines for Life Science and Medical Research Involving Human Subjects and was approved by the Ethical Review Committee of Niigata University of Health and Welfare (Certification No. 18857-220614). The questionnaire explained the purpose of the survey, emphasized that responses were voluntary and that it was not related to school performance, and outlined data disclosure procedures. Written informed consent was obtained from all individual participants included in the study.

## Results

### Response rate and characteristics of the parents

Of the total 418 pairs from which the responses were collected, 85 were excluded because of the missing answer, either by the student or parent. Finally, 333 responses were analyzed. The survey response rate for this study was 49.1% (418 of the 851 pairs collected), and the valid response rate was 79.7%. The 79.0% of the parents who responded to the questionnaire were female, and 70.0% were in their 40 s (Table 1).

**Table 1:** Characteristics of parents who participated in the survey

Age	Sex			Total (%)
	Male	Female	Others	
20–29	1	1	1	3 (0.9)
30–39	1	32	3	36 (10.8)
40–49	29	187	17	233 (70.0)
50–59	5	42	10	57 (17.1)
over 60	2	0	0	2 (0.6)
other	0	1	1	2 (0.6)
Total (%)	38 (11.4)	263 (79.0)	32 (9.6)	333

### Knowledge about kidney function

The most recognized function of the kidneys for both the students and their parents was “removing waste products from the body as urine,” which was understood by 76.3% and 90.1%, respectively (Table 2). Furthermore, 22.2% and 40.5%, respectively, understood that “the kidney regulates the amount of water and minerals in the body,” whereas 21.9% and 64.0%, respectively, understood that “once kidney function declines, it is difficult to return to normal.” Both students and their parents had insufficient knowledge of “blood pressure, hormones related to hematopoiesis,” and “vitamin D activation.” Among the students, 22.5% reported that they did not know any of the items.

**Table 2:** Knowledge of the function of the kidneys (multiple answers allowed)

	Junior high school student (*n* = 333)	Parent (*n* = 333)
	*n* (%)	*n* (%)
Eliminate body wastes as urine	254 (76.3)	300 (90.1)
Secrete hormones that regulate blood pressure	24 (7.2)	60 (18.0)
Secrete blood-forming hormones	22 (6.6)	49 (14.7)
Regulates the amount of water and minerals in the body	74 (22.2)	135 (40.5)
Activates vitamin D, which enhances calcium absorption	10 (3.0)	33 (9.9)
Once kidney function declines, it is difficult to return to normal	73 (21.9)	213 (64.0)
I know of none of these	75 (22.5)	20 (6.0)

The median (interquartile range [IQR]) for the total number of cognitive items related to renal function was one item (1–1) for students and two items (1–2) for parents. The number of cognitive items for students and parents showed a significant positive correlation (ρ = 0.172, *p* < 0.001, 1-β = 0.977) (Table 3).

**Table 3:** Number of items known about the kidney functions

Number of items	Junior high school students	Parents	Differences		
	*n* (%)	n (%)	ρ	*P*-value	Power (1-β)
0	75 (22.5)	20 (6.0)			
1	135 (40.5)	86 (25.8)			
2	74 (22.2)	103 (30.9)			
3	30 (9.0)	65 (19.5)	0.172	0.001	0.977
4	13 (3.9)	17 (5.1)			
5	4 (1.2)	17 (5.1)			
6	2 (0.6)	25 (7.5)			
Median (IQR)	1 (1–1)	2 (1–2)			

### Knowledge of CKD

The 2.4% and 16.5% of the students and their parents, respectively, knew what CKD was, whereas 18.9% and 45.3%, respectively, only knew the name of the disease, and 78.7% and 38.1%, respectively, did not (Table 4). The students and parents knowledge of CKD was associated (χ2(1) = 12.977, *p* < 0.001, ES:φ = 0.197, 1-β = 0.99) (Table 5).

**Table 4:** Knowledge of CKD

	Junior high school student (*n* = 333)	Parent (*n* = 333)
	n (%)	n (%)
People who knew CKD	8 (2.4)	55 (16.5)
People who knew only by name	63 (18.9)	151 (45.3)
People who did not know CKD	262 (78.7)	127 (38.1)

**Table 5:** Association between children’s and parents’ knowledge of CKD

				Junior high school student		Differences			
				People who know CKD* n = 71	People who did not know CKD *n* = 262	χ2	*p*	ES (φ)	Power (1-β)
Parent	People who knew CKD* *n* = 206	n % expected frequency		57 27.7 43.9	149 72.3 162.1	12.977	< 0.001	0.197	0.977
	People who did not know CKD *n* = 127	n % expected frequency		14 11.0 27.1	113 89.0 99.9				

*The group that knew what kind of disease chronic kidney disease was and the group that knew only the name of the disease were combined into the group that knew about CKD

### Relationship between CKD knowledge and the number of items perceived regarding kidney function

**Fig. 2:**
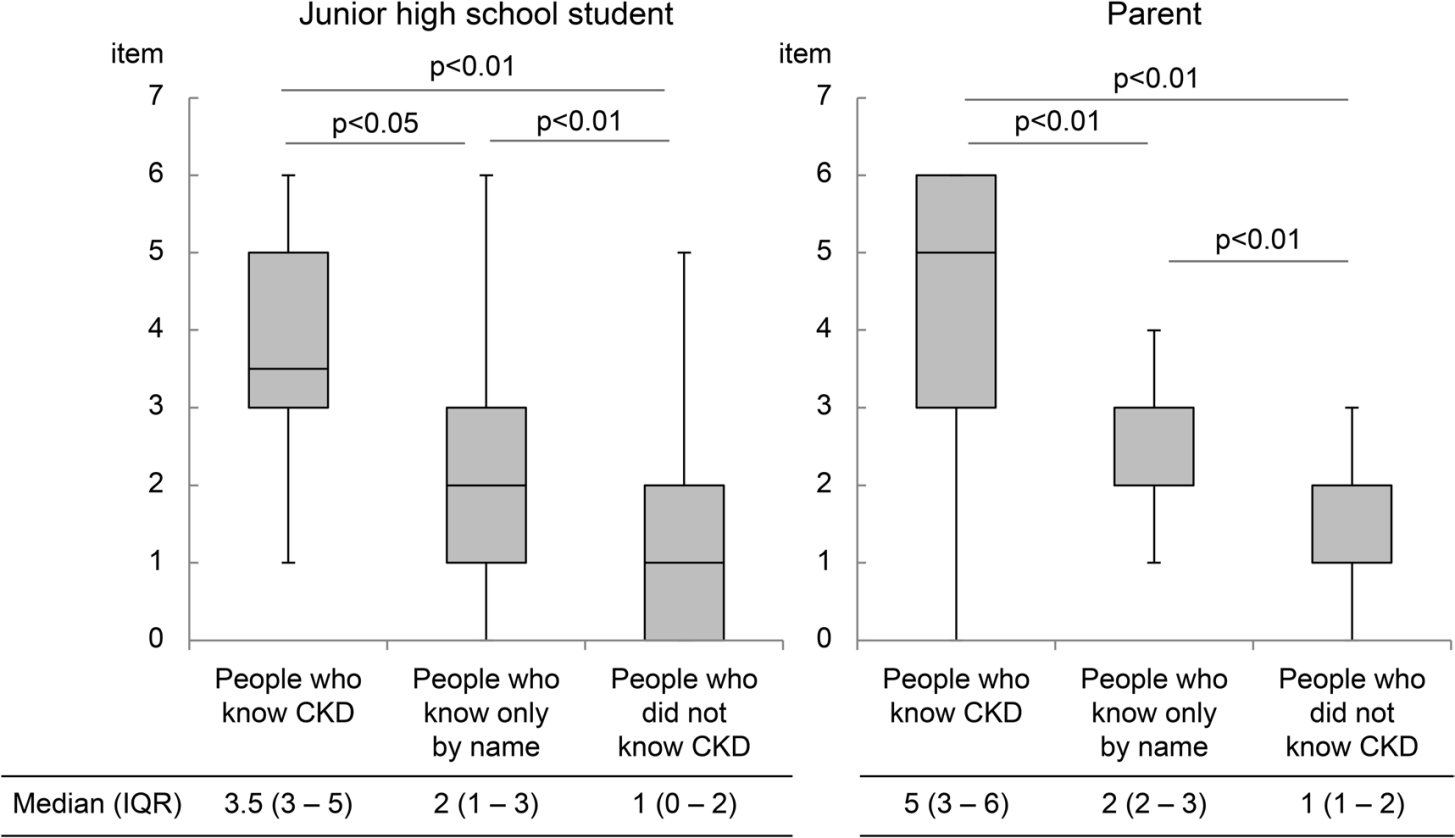
Relationship between CKD knowledge and the number of items perceived regarding kidney function. Increased knowledge of CKD among both junior high school students and their parents is correlated with improved understanding about kidney function. Abbreviation: CKD: chronic kidney disease

The median (IQR) number of cognitive items related to kidney function was 3.5 (3–5) and 5 (3–6) items for students and parents, respectively, in the group that knew what CKD is, 2 (1–3) and 2 (2–3) items, respectively, in the group that only knew the name of the disease, and 1 (0–2) and 1 (1–2) items, respectively, in the group that did not. The group in which both students and parents were familiar with renal function had a significantly better understanding of CKD (p < 0.001 and p < 0.005, respectively) (Fig. 2).

### Parents’ knowledge of CKD, health checkups, urinary protein levels, and eGFR confirmation

In terms of CKD knowledge and health checkup receipt, 89.1%, 80.8%, and 81.1% of the groups who knew about CKD knew only the name of the disease and did not know about CKD, respectively, had health checkups. Notably, medical examination behaviors and CKD knowledge were not associated.

The knowledge of CKD and urinary protein status among the groups was 94.5%, 86.1%, and 64.6%, respectively. Similarly, knowledge of CKD and eGFR status was 50.9%, 14.6%, and 6.4%, respectively. The group with knowledge of CKD was significantly more likely to check urinary protein and eGFR, respectively (χ2(1) = 29.025, *p* < 0.001, ES: Cramer’s V = 0.209, 1-β = 0.880, χ2(1) = 54.846, *p* < 0.001, ES: Cramer’s V = 0.287, 1-β = 0.994) (Table 6).

**Table 6:** Parents’ knowledge of CKD and health checkups

**CKD** **awareness** **level**		**Health checkup**			**Differences**		**Urine protein**			**Differences**				**eGFR**			**Differences**			
		**Annual checkup** *n* = 274	**Every few years** *n* = 47	**Not received** *n* = 12	χ2	*p*	**Checked results** *n* = 264	**Not checked** *n* = 61	**Unknown** *n* = 8	χ2	*p*	Cramer’s *V*	Power (1-β)	**Checked results** *n* = 58	**Not checked** *n* = 124	**Unknown** *n* = 151	χ2	*p*	Cramer’s *V*	Power (1-β)
**People who** **knew CKD**	n %	49 89.1	5 9.1	1 1.8	2.660	0.616	52 94.5	3 5.5	0 0.0	29.025	< 0.001	0.209	0.880	28 50.9	13 23.6	14 25.5	54.846	< 0.001	0.287	0.994
	expected frequency adjusted residual	45.3 1.4	7.8 -1.2	2.0 -0.8			43.6 3.1	10.1 -2.7	1.3 -1.3					9.6 7.2	20.5 -2.3	24.9 -3.2				
**People who** **knew only by name**	n %	122 80.8	24 15.9	5 3.3			130 86.1	18 11.9	3 2.0					22 14.6	59 39.1	70 46.3				
	expected frequency adjusted residual	124.2 -0.6	21.3 0.8	5.4 -0.3			119.7 2.8	27.7 -2.7	3.6 -0.5					26.3 -1.2	56.2 0.6	68.5 0.3				
**People who** **did not know CKD**	n %	103 81.1	18 14.2	6 4.7			82 64.6	40 31.5	5 3.9					8 6.3	52 40.9	67 52.8				
	expected frequency adjusted residual	104.5 -0.4	17.9 0.0	4.6 0.9			100.7 -5.2	23.3 4.9	3.1 1.4					22.1 -4.2	47.3 1.1	57.6 2.1				

## Discussion

Despite the importance of lifelong health education as a measure against lifestyle-related diseases, conventional CKD knowledge surveys have been conducted only for people aged ≥ 20 years and not for the age group in which lifestyle-related disease studies are being introduced. This study clarified the association between the knowledge about CKD among junior high school students (14–15 years old) and their parents, the current state of education on CKD, and the level of awareness among students and their parents. The study revealed four main findings.

First, both the students and their parents had limited knowledge of kidney function and CKD. Their understanding of kidney function was limited to the excretion of waste products. In Japanese schools, the only written information about kidney function is the “excretion of waste products and weakening of kidney function, which causes waste products to accumulate and become life-threatening [19].” In addition, opportunities to learn about CKD other than lifestyle-related diseases, such as heart disease, diabetes, obesity, stroke, periodontal disease, cancer, and hypertension, remain limited [20]. The current low level of understanding of kidney function and CKD among both students and parents may be related to a lack of learning opportunities in educational programs.

Second, both the students and parents in the group who knew about CKD had a better understanding of kidney function. This group was a highly health-literate group that obtained the information on their own, even though they did not learn anything related to CKD in school. Learning, experiences, and environments outside of school may have influenced the differences in knowledge observed in this study. Improving health literacy is important for the promotion and awareness of CKD [21]. Building a social system is necessary for improving health literacy and creating a system in which health information can be obtained naturally. In school education, information is readily available, and effective learning is expected through peer education [22]. School-based education on other diseases and lifestyle habits has been reported to improve students’ knowledge and awareness [23, 24]. In addition, various tools and methods can be effective in health education for adolescents [25]. Adding CKD information to current education could increase awareness of CKD.

Third, the results of this study showed a correlation between the number of perceived kidney function items for junior high school students and their parents. There have been several reports on the reciprocal influence of children and their parents. The health literacy of youth is influenced by parents’ social factors [26]. Conversely, education provided to children can also impact their parents. For example, health education on hypertension for students improved their parent’s knowledge [27]. In this study, children received education at school and reviewed it at home while communicating with their parents. As a result, parental awareness increased, leading to a medical examination. It is possible that the knowledge association observed in our study may have occurred because this generation of children and parents influenced each other. On the other hand, another study reported that adolescents’ habitual nutritional intake was not directly associated with mothers’ nutritional knowledge [28], and that children of parents with higher levels of nutritional knowledge are more likely to be overweight or obese [29]. In these studies, the mutual understanding between children and parents was not evaluated, and it is possible that a lack of communication did not lead to knowledge retention or changes in their behavior.

Finally, no association was observed between knowledge of CKD and health-checkups behavior among the parents. This is possible to mean, they were just taking health checkups even though little interest in CKD. On the other hand, knowledge of CKD was associated with urinary protein level and eGFR checking status. In the present study, the group that knew about CKD exhibited a better understanding of kidney function. Understanding organ function is necessary for understanding the disease. Therefore, there is a need for educational opportunities to raise awareness about CKD. Schools should educate children, who can then discuss and share information with their parents at home. This approach may lead to a better understanding of urinary protein and eGFR during health checkups, potentially resulting in earlier detection of CKD.

This study had four limitations. First, the study was conducted only in schools from two areas of Niigata City; therefore, our results cannot be generalized to other areas. CKD promotion and awareness activities may differ depending on local factors, such as health literacy, limiting generalizability. Contrastingly, in the Niigata prefecture, CKD promotion and awareness activities commenced in 2007 [30]; since then, these activities have received an increased level of interest in the area. Nevertheless, the limited knowledge of CKD among the younger generation and the lack of awareness about its early detection need to be addressed in future promotional and educational activities. Second, social background, such as parental occupation and income, may have influenced the responses. In areas with fewer healthcare providers, awareness of CKD is expected to be lower. However, this study was performed in an area with a relatively even distribution of healthcare professionals [31]; selecting a general area may have reduced this bias. Third, although responses to the questionnaire in this study were voluntary, selection bias was possible because those with relatively high health awareness responded to the questionnaire. However, CKD knowledge surveys reported in Japan are often conducted via the Internet, at health checkup sites, and at CKD promotion and awareness events. They are often answered by groups with a high level of interest in health. In this study, the survey was conducted in a school-based setting and included responses from groups that were not actively involved in health promotion, thereby providing a relatively wide range of results despite the limitations of the survey. Finally, the questionnaire was designed to be answered jointly by students and parents, and knowledgeable parents might have provided answers to students; however, this potential influence does not negate the overall finding: knowledge about CKD remains inadequate.

Despite these limitations, this is the first CKD knowledge survey among students and their parents, and the results may provide new insights into CKD awareness. We expect that communication between children and parents will increase CKD knowledge for both, leading to improved awareness of the disease. Thus, future research is needed to determine whether interventions with students improve their CKD knowledge status, change the behavior of students and their parents, and relate knowledge to situations of increased CKD knowledge.

## Conclusion

The study revealed that both junior high school students (14–15 years old) and their parents exhibited insufficient knowledge of renal function, along with an association between knowledge of CKD among students and their parents and an association between the understanding of CKD, urinary protein levels, and eGFR status in the parents. Notably, school-based education has the potential to increase their parents and younger generations’ knowledge of CKD and their understanding of health checkups results. Our results provide new insights into effective methods for future dissemination and awareness.
