# Correction: Adolescents and parents’ knowledge of chronic kidney disease: the potential of school-based education

**DOI:** 10.1007/s10157-025-02627-6

**Published:** 2025-01-23

**Authors:** Junko Nakamura, Ryohei Kaseda, Mizuki Takeuchi, Kou Kitabayashi, Ichiei Narita

**Affiliations:** 1 Division of Clinical Nephrology and Rheumatology, Niigata University Graduate School of Medical and Dental Sciences, 1-757 Asahimachi-Dori, Chuo-ku, Niigata, 951-8510 Japan; 2 Department of Health and Nutrition, Faculty of Health Science, Niigata University of Health and Welfare, 1398 Shimami-Cho, Kita-ku, Niigata, 950-3198 Japan; 3 Department of Nutrition, Shinkohkai Murakami Kinen Hospital, 204-1 Matsuyama, Murakami, 958-0034 Japan; 4 Niigata Institute for Health and Sports Medicine, 67-12 Ceigoro Chuo-ku, Niigata, 950-0933 Japan

**Correction: Clinical and Experimental Nephrology** 10.1007/s10157-024-02574-8

The article “Adolescents and parents’ knowledge of chronic kidney disease: the potential of school-based education”, written by https://link.springer.com/article/10.1007/s10157-024-02574-8#auth-Junko-Nakamura-Aff1-Aff2 Junko Nakamura, https://link.springer.com/article/10.1007/s10157-024-02574-8#auth-Ryohei-Kaseda-Aff1 Ryohei Kaseda, https://link.springer.com/article/10.1007/s10157-024-02574-8#auth-Mizuki-Takeuchi-Aff2 Mizuki Takeuchi, https://link.springer.com/article/10.1007/s10157-024-02574-8#auth-Kou-Kitabayashi-Aff3 Kou Kitabayashi and https://link.springer.com/article/10.1007/s10157-024-02574-8#auth-Ichiei-Narita-Aff4 Ichiei Narita was originally published under exclusive license to Japanese Society of Nephrology. As a result of the subsequent decision to publish the article under the open access model, the article’s copyright notice was changed on 06 January 2025to © The Author(s) 2025 and the article is now distributed under a Creative Commons Attribution 4.0 International License, which permits use, sharing, adaptation, distribution and reproduction in any medium or format, as long as you give appropriate credit to the original author(s) and the source, provide a link to the Creative Commons licence, and indicate if changes were made. The images or other third party material in this article are included in the article’s Creative Commons licence, unless indicated otherwise in a credit line to the material. If material is not included in the article’s Creative Commons licence and your intended use is not permitted by statutory regulation or exceeds the permitted use, you will need to obtain permission directly from the copyright holder. To view a copy of this licence, visit http://creativecommons.org/licenses/by/4.0.

